# Machine Learning Enables Prediction of Cardiac Amyloidosis by Routine Laboratory Parameters: A Proof-of-Concept Study

**DOI:** 10.3390/jcm9051334

**Published:** 2020-05-03

**Authors:** Asan Agibetov, Benjamin Seirer, Theresa-Marie Dachs, Matthias Koschutnik, Daniel Dalos, René Rettl, Franz Duca, Lore Schrutka, Hermine Agis, Renate Kain, Michela Auer-Grumbach, Christina Binder, Julia Mascherbauer, Christian Hengstenberg, Matthias Samwald, Georg Dorffner, Diana Bonderman

**Affiliations:** 1 Section for Artificial Intelligence and Decision Support, Medical University of Vienna, 1090 Vienna, Austria; asan.agibetov@meduniwien.ac.at (A.A.); matthias.samwald@meduniwien.ac.at (M.S.); georg.dorffner@meduniwien.ac.at (G.D.); 2 Division of Cardiology, Medical University of Vienna, Waehringer Guertel 18-20, 1090 Vienna, Austria; n11700881@students.meduniwien.ac.at (B.S.); theresa-marie.dachs@meduniwien.ac.at (T.-M.D.); matthias.koschutnik@meduniwien.ac.at (M.K.); daniel.dalos@meduniwien.ac.at (D.D.); rene.rettl@meduniwien.ac.at (R.R.); franz.duca@meduniwien.ac.at (F.D.); lore.schrutka@meduniwien.ac.at (L.S.); christina.binder@meduniwien.ac.at (C.B.); julia.mascherbauer@meduniwien.ac.at (J.M.); christian.hengstenberg@meduniwien.ac.at (C.H.); 3 Division of Oncology, Medical University of Vienna, 1090 Vienna, Austria; hermine.agis@meduniwien.ac.at; 4 Division of Pathology, Medical University of Vienna, 1090 Vienna, Austria; renate.kain@meduniwien.ac.at; 5 Division of Orthopedics and Traumatology, Medical University of Vienna, 1090 Vienna, Austria; michela.auer-grumbach@meduniwien.ac.at

**Keywords:** heart failure, cardiac amyloidosis, machine learning, artificial intelligence

## Abstract

(1) Background: Cardiac amyloidosis (CA) is a rare and complex condition with poor prognosis. While novel therapies improve outcomes, many affected individuals remain undiagnosed due to a lack of awareness among clinicians. This study was undertaken to develop an expert-independent machine learning (ML) prediction model for CA relying on routinely determined laboratory parameters. (2) Methods: In a first step, we developed baseline linear models based on logistic regression. In a second step, we used an ML algorithm based on gradient tree boosting to improve our linear prediction model, and to perform non-linear prediction. Then, we compared the performance of all diagnostic algorithms. All prediction models were developed on a training cohort, consisting of patients with proven CA (positive cases, *n* = 121) and amyloidosis-unrelated heart failure (HF) patients (negative cases, *n* = 415). Performances of all prediction models were evaluated on a separate prognostic validation cohort with 37 CA-positive and 124 CA-negative patients. (3) Results: Our best model, based on gradient-boosted ensembles of decision trees, achieved an area under the receiver operating characteristic curve (ROC AUC) score of 0.86, with sensitivity and specificity of 89.2% and 78.2%, respectively. The best linear model had an ROC AUC score of 0.75, with sensitivity and specificity of 84.6 and 71.7, respectively. (4) Conclusions: Our work demonstrates that ML makes it possible to utilize basic laboratory parameters to generate a distinct CA-related HF profile compared with CA-unrelated HF patients. This proof-of-concept study opens a potential new avenue in the diagnostic workup of CA and may assist physicians in clinical reasoning.

## 1. Introduction

While heart failure (HF) syndrome is a common condition, in particular among elderly patients [1], the spectrum of underlying entities necessitating specific treatments is widely spread. The spectrum covers rather prevalent conditions, such as ischemic heart disease with reduced left ventricular ejection fraction (EF), but also rare and underexplored conditions that may well present with preserved left ventricular EF. In parallel, there is a broad awareness of more prevalent HF types among physicians and health care providers, while rare HF types often remain undiagnosed or diagnosis may be delayed for many years. In fact, rare diseases are often diagnosed in expert centers, which usually stand at the end of a very long patient journey.

Cardiac amyloidosis (CA) is one of these rare and complex conditions with poor prognosis ([2,3]). The two predominant subtypes of amyloid deposition disease affecting the heart are (1) transthyretin and (2) light-chain amyloidosis. Both CA subtypes have recently gained attention, as novel disease-modifying therapeutic approaches have become available ([4,5,6]). However, a meaningful supply of potentially life-saving therapies requires accurate and timely diagnosis, before the occurrence of irreversible organ damage.

One approach to overcome the incongruence between the availability of patient-tailored therapies and the lack of disease awareness among physicians and health care providers is the use of automated diagnostic algorithms that are built on broadly available and routinely obtained patient data. The present study was driven by the conception that, irrespective of geographical and health care system idiosyncrasies, a palette of routine laboratory parameters is usually obtained from patients presenting with HF symptoms, as a baseline evaluation. We hypothesized here that patients with CA-associated HF display a distinct laboratory parameter pattern as compared to patients presenting with other HF entities, and that machine-learning algorithms may help to identify these patterns. To that end, we have prospectively enrolled patients with proven CA, both the transthyretin and light-chain subtypes, and non-related HF phenotypes, who served as controls. Routinely available laboratory parameters, excluding heart-specific biomarkers, such as natriuretic peptides, were used to develop an automated pipeline for parameter selection, in order to build the best-performing prediction model. In the following, we present cross-validated data and data that have been validated in an unrelated prospective cohort that support this novel expert-independent diagnostic approach for CA.

## 2. Materials and Methods

### 2.1. Setting and Study Design

This was a single-center study that was performed within the frames of a prospective HF registry, and all participants gave their written informed consent (EK# 796/2010). Our center is located at the Medical University of Vienna, which is a tertiary care center with a high-volume cardiac catheterization unit and a high-volume cardiac transplantation program. Moreover, we are part of the European Reference Network for Amyloidosis and a national referral center for patients with heart failure and preserved ejection fraction (HFpEF).

### 2.2. Diagnostic Procedures

#### 2.2.1. Cardiac Amyloidosis

Transthyretin CA was diagnosed by endomyocardial biopsy before 2016. Following the publication of a non-invasive diagnostic algorithm by Gillmore et al. [7], endomyocardial biopsy was only performed when non-invasive diagnostic results were ambiguous or unclear. All patients with transthyretin CA underwent genetic testing to differentiate between wild-type and hereditary disease.

Light-chain CA was diagnosed by endomyocardial biopsy using Congo red staining and immunohistochemistry to categorize the amyloidogenic precursor protein. Alternatively, light-chain CA was diagnosed when extra-myocardial biopsy was positive for light chains and transthoracic echocardiography (TTE) or cardiac magnetic resonance (CMR) imaging showed signs of left ventricular hypertrophy with an interventricular septum thickness > 12 mm and laboratory testing confirmed elevated cardiac biomarkers [8].

Patients with interventricular septum thickness by TTE ≥ 15mm and/or CA red flag signs, such as a history of carpal tunnel syndrome, underwent CA-specific diagnostic procedures independent from CMR results. All other patients were referred to CMR, including T1 mapping (T1 weighted image (also referred to as T1WI or the “spin-lattice” relaxation time) is one of the basic pulse sequences in MRI and demonstrates differences in the T1 relaxation times of tissues.) as a first diagnostic step. Only those with CMR-based suspicion of CA underwent CA-specific diagnostic tests. 

#### 2.2.2. Amyloidosis-Unrelated Heart Failure

The spectrum of controls included patients with HFpEF or HF with reduced EF (HFrEF), valvular heart disease, cardiac sarcoidosis, and hypertrophic cardiomyopathy with or without left ventricular outflow tract obstruction, as well as other rare HF conditions. For respective diagnoses, all patients underwent TTE and CMR. In ambiguous cases, right heart catheter was performed. In addition, CA was excluded in all participants, as suggested by CMR and/or the algorithm by Gillmore et al. [7]. Beyond structural abnormalities of the heart, patients were symptomatic with shortness of breath and displayed elevated natriuretic peptides, thus fulfilling the diagnostic criteria for HF [1]. However, classical HF biomarkers, such as natriuretic peptides, were not included in the statistical analysis.

### 2.3. Statistical Procedures

To analyze differences between amyloidosis and control patients, the two-sample t-test was used for continuous variables, and Chi-squared and Fischer’s exact tests were used for binary variables. All *p*-values were adjusted with the Benjamini–Hochberg procedure (false discovery rate alpha = 0.05).

In this work, we used an ensemble machine learning technique of gradient-boosted classification [9] and regression trees (CART) for two purposes: (1) ranking laboratory parameters by their relative importance for final prediction of CA [10], and (2) fully-automated prediction of CA. In our work, we used a specific implementation of gradient boosting for CARTs – XGBoost (the name of the Python library) [11].

In the development of linear logistic regression models, we used imputation techniques to deal with missing values. For variable selection, we used a logistic regression model with L1 regularization (Lasso [12]). For all our experiments, we performed optimization of our prediction algorithms on our training dataset (training patient cohort) with stratified cross-validation, where cross-validation folds preserve class sample ratio. The performance of all models was measured with area under receiver-operating characteristic curve (ROC AUC) on the holdout test dataset (prospective validation cohort). We have chosen ROC AUC score because our classification problem was an imbalanced one, which means that there were more negative samples than positives, with a CA prevalence rate of 23%. To compute the diagnostic accuracy, including sensitivity, specificity, positive prediction value (PPV), negative prediction value (NPV) and false omission rate (FOR), we used the Youden J statistic [13], which optimizes the operating point (true positive rate and false positive rate) of a prediction model from the ROC curve.

## 3. Results

### 3.1. Patient Baseline Characteristics

Between December 2010 and October 2018, a total of 536 consecutive patients were registered and their routine laboratory parameters served as training dataset for all statistical modeling experiments. Their detailed clinical baseline characteristics are displayed in Table 1. In brief, 121 (22.5%) were diagnosed with CA-associated HF (positive cases) and the remaining 415 patients with unrelated HF types (negative cases). Among negatives, the predominant condition was HFpEF (*n* = 331), 32 patients were diagnosed with HFrEF, 24 patients had valvular heart disease, 14 patients had cardiac sarcoidosis, 6 patients had hypertrophic cardiomyopathy, and 8 patients suffered from other rare HF conditions. In general, amyloidosis patients were older than control patients (*p* = 0.004). They were predominantly male (71.9%), while controls were predominantly female (59.8%, *p* < 0.001). Of note, CA-related HF patients were in rather advanced disease stages when compared to controls, as documented by higher N-terminal prohormone of brain natriuretic peptide (NT-proBNP) levels (median NT-proBNP in pg/mL: 3132.5 [1343.8, 6994.2] in CA patients versus 680.0 [226.1, 1621.0] in controls, *p* < 0.001)).

For validation purposes, a subsequent prospective cohort (*n* = 160) consisting of 36 (22.5%) amyloidosis patients and 124 HF controls was enrolled between November 2018 and June 2019. The majority of negatives (*n* = 57) had HFpEF, 24 had valvular heart disease, 16 had HFrEF, 15 had cardiac sarcoidosis, 4 had hypertrophic cardiomyopathy, and 8 had other rare heart failures. Table 2 provides a summary of the baseline characteristics of the validation cohort.

In our study, we analyzed data from 63 patients with Amyloid light-chain amyloidosis AL (40%), 82 with wild-type Amyloid Transthyretine (ATTR) (52%), and 12 patients with variant ATTR (8%) (Table S2).

### 3.2. Development of the Diagnostic Algorithm with Imputation of Missing Values

At baseline, 62 routine laboratory parameters, including clinical chemistry parameters, blood cell count and coagulation parameters, were available for algorithm development. Although a certain core of parameters was consistently determined in each patient, such as liver and kidney function parameters, others had a high incidence of missing values.

**Figure 1:**
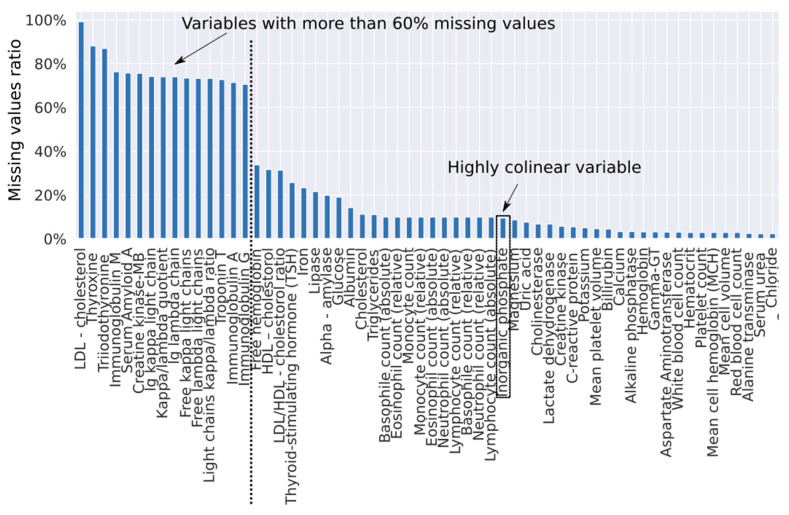
Ratio of missing values per parameter. The truncated set of parameters does not include parameters with a missing ratio >= 0.6 and does not include a highly collinear “Inorganic phosphate” parameter.

In a first step, we developed a diagnostic algorithm based on logistic regression with iterative imputation of missing values. Before training the diagnostic algorithm, we excluded 16 parameters with a high missing values ratio (cut-off at 60% missing ratio; 15 parameters) and highly collinear parameters (cut-off at 0.98 Pearson correlation; 4 parameters); 13 parameters had both a high missing values ratio and a high collinearity index (Figure 1).

After the exclusion of 16 parameters, we considered 46 laboratory parameters. In the rest of the manuscript, we refer to these 46 parameters as the truncated set of parameters. When we used a logistic regression model with L1 regularization, we found that, out of 46 truncated parameters, only 25 were important for the prediction as they had non-zero coefficients. When evaluated on the independent test set, the ROC AUC score of this model was 0.58, with sensitivity, specificity, PPV, and NPV of 67.6%, 53.2%, 30.0%, and 84.6%, respectively. Resulting from NPV calculations, the false omission rate (FOR) for this model was 15.4%.

### 3.3. Improving the Diagnostic Algorithm with Machine Learning

**Figure 2:**
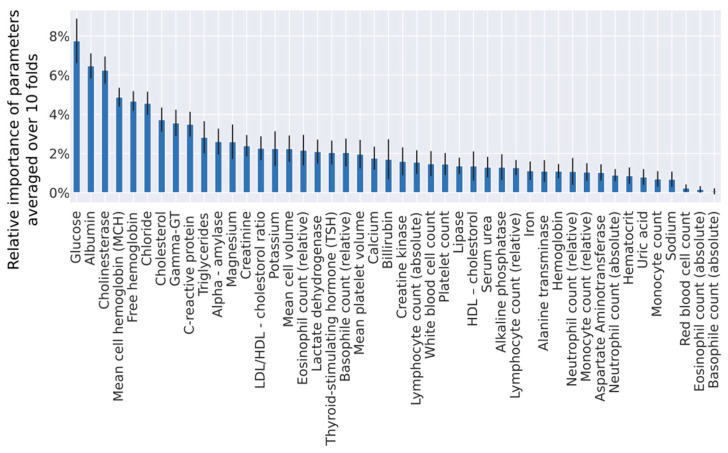
Relative importance of parameters from the truncated set, measured by the XGBoost algorithm. Means and standard deviations computed from a 10-fold cross-validation are displayed in this graph.

To improve our diagnostic algorithm, we used an XGBoost algorithm (gradient-boosted tree ensembles) to determine the relative importance of each variable towards the final prediction. To be robust against the non-determinism, we fitted this algorithm on training data in a 10-fold cross-validation loop (Figure 2). We found that we needed at least 91% of variables to reach 99% of cumulative importance, which means that it is impossible to select only a few variables that would explain most of data variability. To take into account the broad cumulative importance curve, we took averaged contribution factors obtained from 10 folds to rank all variables. This averaged ranking was then used to identify the top n important variables in our prediction model. To exactly determine how many of these ranked parameters we need for a better diagnostic algorithm, we iteratively trained logistic regression models by gradually including ordered predictor variables and measuring the performance of each model on the holdout test set.

**Figure 3:**
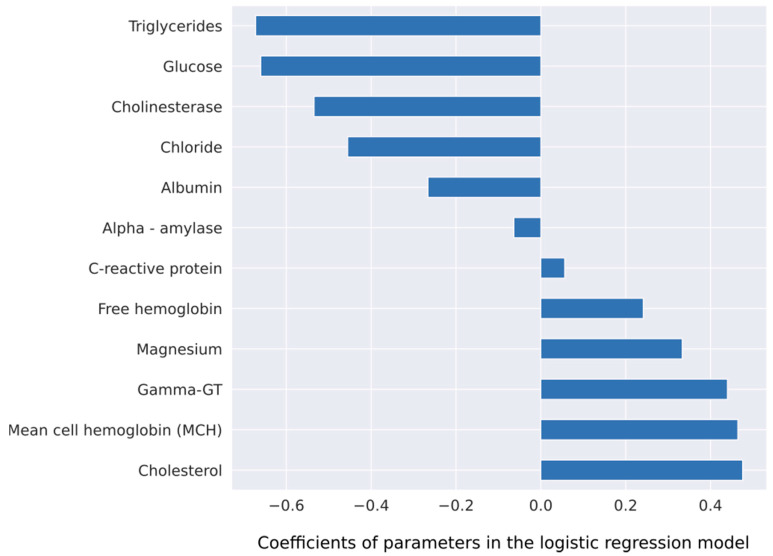
Coefficients of a logistic regression model for the outcome prediction. This model uses 12 parameters, which were identified by the XGBoost algorithm.

The overall best model was obtained with the 12 most important variables (Figure 3) and a case deletion strategy for missing values (event per ratio rate 66/12). Performance of this model had a diagnostic accuracy of 0.75 ROC AUC, with sensitivity, specificity, PPV, and NPV of 84.6%, 71.7%, 33.3%, 96.6% (FOR 3.4%), respectively.

### 3.4. Fully Automated Machine Learning Diagnostic Algorithm

Ensemble of gradient-boosted decision trees can directly be used as a prediction model. The biggest advantage of using this powerful algorithm is its ability to handle missing data and automatically discover non-linear interactions among the parameters. Because it can handle missing data, we trained this algorithm on all parameters. On the independent test set, the performance of a fully automated algorithm had a ROC AUC score of 0.86, and sensitivity, specificity, PPV and NPV of 89.2%, 78.2%, 55.0% and 96.0% (FOR 3.9%), respectively. A fully automatic prediction non-linear model trained on the full set of parameters displayed better performance than a linear model trained on the truncated set of parameters with an increase of 0.11 in the ROC AUC score, 4.6% in sensitivity, and 6.5% in specificity. We compared this model with an ensemble model trained on the truncated set of parameters; the performance on the holdout test set was worse than on the full set of parameters: 0.66 ROC AUC, with sensitivity, specificity, PPV and NPV of 67.6%, 70.2%, 40.3% and 87.9% (FOR 12.1%), respectively. Diagnostic performances of all models are summarized in Table 3.

## 4. Discussion

Patients with CA-driven HF display symptoms that are indistinguishable from those reported by patients with other HF phenotypes. Moreover, classical diagnostic tools that are routinely used for HF diagnosis and its etiology, such as cardiac biomarkers, electrocardiographic markers and imaging techniques, require advanced expert knowledge to discern between amyloid-driven HF and non-amyloid-associated HF [1]. A combination of two diagnostic key tools, i.e., paraprotein detection and DPD scan, suffice in a vast majority of cases to diagnose or exclude CA [7]. However, an inclusion of this diagnostic step in the work-up of HF patients requires disease awareness, expert knowledge, and potential application of diagnostic algorithms that are again based on rather advanced diagnostic techniques ([14,15]).

In light of recent advances in data science and applications of ML-based algorithms, we hypothesized here that HF patients with CA might display distinct biomarker patterns as compared to non-CA HF patients that are detectable by intelligent statistical approaches. We were particularly interested in biomarkers that are routinely and broadly used in the work-up of cardiac or even internal ailment patients and do not require specific expert skills, such as acquisition, measurement or interpretation. We therefore focused on routine laboratory parameters, based upon which we built two alternative prediction models. The first model utilized twelve routine laboratory parameters, via a simple and interpretable linear prediction model (logistic regression) that was backed by a complex machine learning algorithm (ensemble of decision trees) for variable selection. This model reached a diagnostic prediction accuracy in an unrelated prospective cohort consisting of HF patients with and without CA of 0.75 ROC AUC, with sensitivity 84.6%, specificity 71.7%, positive predictive value 47.1%, and negative predictive value 96.6% (FOR 3.4%). Even more convincing was the prediction model proposed by a black-box algorithm (non-linear complex machine learning model) that could be confirmed by the results obtained from the unrelated validation cohort (0.86 ROC AUC score, with sensitivity, specificity, PPV and NPV of 89.2%, 78.2%, 55.0% and 96.0% (FOR 3.9%), respectively).

With respect to the choice of parameters for respective prediction model construction, there was a clear difference between the two statistical approaches. The logistic regression model identified lower serum levels of known cardiovascular risk factors, such as serum triglycerides (*p* = 0.008) and blood glucose (*p* = 0.008) as associated with CA (Table S1). From a clinical perspective, one might argue that patients with amyloidosis-driven HF develop HF despite the absence of classical cardiovascular risk factors that are held responsible for ischemic heart disease and also HFpEF. The other parameter cluster that was identified to differentiate between amyloidosis-driven HF and other HF types may be considered as indicative for liver congestion in the context of right ventricular dysfunction. In fact, serum gamma-GT was identified as a predictor of adverse outcome in HF, with reduced as well as with preserved EF [16]. Lower serum albumin levels and cholinesterase levels as indicators of impaired liver synthesis function may also be attributed to more advanced HF at diagnosis as compared to other HF phenotypes. This finding may well be explained by the notion of delayed diagnosis of CA as compared to other HF phenotypes [17]. Taken together, the diagnostic algorithm suggested by the logistic regression approach may pin down HF patients with a less pronounced serum-based cardiovascular risk profile but more advanced HF in the sense of more pronounced laboratory biomarkers of liver congestion. By contrast, the ML-based algorithm may better deal with missing values [10]. Therefore, CA-specific parameters that were only available in a limited patient group were integrated in the black-box algorithm despite a significant number of missing values. In this algorithm, Troponin T and presence of amyloidogenic paraprotein, i.e., elevated free lambda light chains and an elevated kappa/lambda ratio. While elevated Troponin T levels are a hallmark of CA [18], both ATTR- and AL-free light chains are pathognomonic for AL amyloidosis. With respect to the other laboratory biomarkers selected by the black box, there was an overlap with parameters selected by the logistic regression model. In fact, parameters reflecting lower cardiovascular risk as well as parameters most likely reflecting liver congestion were among the strongest predictors of amyloidosis-associated HF.

Findings of the present study may well change the clinical routine, at least in our center. First, we have established a web-based calculator (http://amyloidosis.plumdeq.xyz) that will help to differentiate amyloidosis and non-amyloidosis-driven HF. Although not yet validated externally, the application can be used to assist with regards to clinical decision-making. Second, distinct laboratory biomarker profiles of CA versus non-CA HF may be incorporated into physicians’ clinical reasoning and decision-making. Third, other laboratory biomarkers that have been linked with amyloidosis-driven HF by both statistical approaches, such as markers associated with red blood cells and inflammation, could also influence clinical reasoning, although the pathophysiological association with CA remains to be elucidated.

The sensitivity of both models is high, which means that these models could be used for positive CA patient screening. The proposed linear model would discover 84.6% of positive patients, and the non-linear AI model would find 89.2%. However, both models are slightly worse at excluding non-amyloid patients than at detecting positives, with a specificity of 71.7% for the linear, and 78.2% for the non-linear, respectively. Therefore, such automated prediction models should only be used for screening CA patients followed by other confirmatory tests. As is well known, in cases of low disease prevalence, even a nearly perfect prediction algorithm with a sensitivity and specificity of 95% would still give many false positives (only 16% of positively screened patients would be true positives, if the disease prevalence is 1%). Considering that CA is a rare HF condition, the proposed prediction models should not be applied to general populations with an extremely low disease prevalence (<< 1%). We tested our algorithm on pre-selected cohorts manifesting symptoms of HF with an amyloidosis prevalence of 23%, which is relatively high for a rare disease with a prevalence of 1 in 10,000.

### Limitations

A major limitation of the present study is that both algorithms are exclusively applicable in our center. However, this study was designed as a proof of concept rather than a generalizable prototype. To the best of our knowledge, this is the first study that uses routine laboratory parameters to identify disease-specific patterns, in order to build diagnostic prediction models.

Due to the limited sample sizes of positive patients, we have not focused primarily on the development of patient profiles for two major types of amyloidosis, ATTR and AL. However, development of patient profiles for specific types of amyloidosis might be a very interesting research avenue for future work, bearing in mind that it would require reasonable sample sizes for each amyloidosis type. Likewise, due to the fact that most CA patients were in advanced HF stages, our algorithm will fail to identify individuals with early disease. However, our findings may fuel future research attempting to do so.

Another limitation of the study is a gender mismatch between patients and controls. While it is known that males have a higher incidence of amyloidosis—which is also reflected in both the training and the validation cohort—we had slightly more female patients among controls. However, when we excluded a significant number of female patients from both cohorts, balancing the gender distribution to 70% males and 30% females, we obtained slightly worse accuracy results (ROC AUC dropped from 0.86 to 0.80). The drop in accuracy could be associated with a true difference in baseline characteristics of patients of different genders, but it can also be attributed to the reduced sample size.

In a broader sense, using artificial intelligence (AI)-based web calculators may raise ethical issues. Theoretically, an AI algorithm looks at all data, instead of focusing on a specific patient profile. For instance, AI could notify patients about a high risk of cancerogenous pathways, although patient data were initially scanned for amyloidosis or other disease. This may be categorized as a violation of patient privacy. Therefore, in our work we only considered study participants who had presented with symptoms of HF in a designated HF unit. We believe that in this specific clinical scenario ethical issues are less critical.

Furthermore, we did not attempt to improve the diagnostic accuracy of prediction models by adding other non-invasively obtained biomarkers, such as electrocardiography, although its value for an automated reader-independent diagnosis of a reduced left ventricular EF has recently been shown [19]. This could be subject of future studies.

## 5. Conclusions

The conceptual aim of this study was to improve the diagnosis of CA among patients presenting with symptoms of HF. Relying on routinely determined laboratory parameters and ML algorithms, we could build two alternative prediction models that were able to identify affected patients with a high diagnostic accuracy. External validation studies in a multi-center setting are warranted in order to obtain generalizable results. Overall, our diagnostic principle may be useful in other clinical settings, beyond CA and HF. This should be investigated in future studies.

## Figures and Tables

**Table 1:** Baseline characteristics for the training cohort.

	Amyloidosis-Unrelated HF (*n* = 415)	Amyloidosis-Related HF (*n* = 121)	*p*-Value
**Variable, median (Q1–Q3)]**			
Age, years	69.0 (58.0, 75.0)	73.0 (62.0, 78.0)	0.004
NT-proBNP, pg/mL	680.0 (226.1, 1621.0)	3132.5 (1343.8, 6994.2)	< 0.001
**Variable, mean (SD)**			
Body mass index, kg/m^2^	29.8 (6.6)	26.0 (4.1)	< 0.001
**Variable, *n* (%)**			
Gender, males	167 (40.2)	87 (71.9)	< 0.001
Coronary artery disease	112 (27.7)	23 (19.5)	0.114
Atrial fibrillation	173 (42.6)	47 (39.5)	0.679
Arterial hypertension	346 (83.8)	67 (57.3)	< 0.001
Diabetes mellitus	118 (28.6)	11 (9.2)	< 0.001
Hyperlipidemia	220 (54.3)	26 (22.4)	< 0.001
MRA	104 (26.3)	51 (45.1)	0.001
Calcium channel blocker	93 (23.1)	9 (7.7)	0.001
Beta-blocker	278 (68.6)	58 (52.7)	0.004
Diuretics	213 (53.8)	80 (70.2)	0.004
ACEI/ARB	253 (62.5)	49 (43.8)	0.001
Oral anticoagulant	184 (45.7)	54 (47.0)	0.888
Statin	181 (44.6)	29 (25.0)	0.001

NT-proBNP, N-terminal prohormone of brain natriuretic peptide; MRA, mineralocorticoid receptor antagonist; ACEI/ARB, angiotensin-converting enzyme inhibitors/angiotensin receptor blocker.

**Table 2:** Baseline characteristics for the prospective validation cohort.

	Amyloidosis-Unrelated HF (*n* = 124)	Amyloidosis-Related HF (*n* = 36)	*p*-Value
**Variable, median (Q1-Q3)**			
Age, years	75.0 (69.0,78.0)	78.0 (71.8,83.0)	0.066
NT-proBNP, pg/mL	851.0 (372.1,1939.0)	2568.5 (1543.8,4334.0)	<.001
**Variable, mean (SD)**			
Body mass index, kg/m^2^	29.5 (5.5)	26.0 (4.5)	0.003
**Variable, *n* (%)**			
Gender, males	36 (29.5)	28 (77.8)	<0.001
Coronary artery disease	33 (26.8)	16 (44.4)	0.222
Atrial fibrillation	69 (58.0)	18 (50.0)	0.638
Arterial hypertension	110 (93.2)	25 (69.4)	0.003
Diabetes mellitus	36 (30.8)	8 (22.2)	0.638
Hyperlipidemia	63 (52.9)	16 (44.4)	0.638
MRA	60 (50.4)	19 (52.8)	0.954
Calcium channel blocker	26 (21.7)	4 (11.1)	0.534
Beta-blocker	85 (70.8)	18 (50.0)	0.127
Diuretics	80 (67.8)	28 (77.8)	0.634
ACEI/ARB	88 (73.3)	23 (63.9)	0.634
Oral anticoagulant	75 (61.0)	19 (52.8)	0.638
Statin	59 (50.0)	16 (44.4)	0.804

NT-proBNP, N-terminal prohormone of brain natriuretic peptide; MRA, mineralocorticoid receptor antagonist; ACEI/ARB, angiotensin-converting enzyme inhibitors/angiotensin receptor blocker.

**Table 3:** Diagnostic performance of prediction models.

Model	ROC AUC	Sensitivity %	Specificity %	PPV %	NPV (FOR) %
LR-L1	0.58	67.6	53.2	30.0	84.6 (15.4)
LR-XGBoost	0.75	84.6	71.7	33.3	**96.6 (3.4)**
XGBoost-62	**0.86**	**89.2**	**78.2**	**55.0**	96.1 (3.9)
XGBoost-46	0.66	67.6	70.2	40.3	87.9 (12.1)

LR-L1—logistic regression with lasso, LR-XGBoost—logistic regression on most important variables identified by XGBoost. XGBoost-62 and XGBoost-46—XGBoost algorithm trained on 62 and 46 parameters, respectively. ROC AUC—area under receiver operating curve, PPV—positive prediction value, NPV—negative prediction value, FOR—false omission rate. Bold indicates best results.

## Supplementary Materials

The following are available online at https://www.mdpi.com/2077-0383/9/5/1334/s1, Table S1: Lab values characteristics for the training cohort. Table S2: Baseline characteristics for all cardiac amyloidosis patients.
