# Supplementary material for: Machine Learning Enables Prediction of Cardiac Amyloidosis by Routine Laboratory Parameters: A Proof-of-Concept Study
Source: J Clin Med. 2020 May 3;9(5):1334. doi: 10.3390/jcm9051334 (PMC7290438; doi:10.3390/jcm9051334)
Supplement: Supplementary file 1 [file jcm-09-01334-s001.zip › train-cohort-lab-values.pdf]

		Grouped by label				
		Missing	0.0	1.0	P-Value (adjusted)	Test
n			415	120		
Cholinesterase, median [Q1,Q3]		39	7.4 [6.3,8.7]	6.0 [4.9,7.4]	<0.001	Kruskal-Wallis
Gamma-GT, median [Q1,Q3]		18	32.0 [20.0,56.8]	64.0 [30.0,135.5]	<0.001	Kruskal-Wallis
Creatinine, median [Q1,Q3]		15	1.0 [0.8,1.2]	1.1 [1.0,1.5]	<0.001	Kruskal-Wallis
Chloride, median [Q1,Q3]		15	103.0 [100.0,105.0]	101.0 [98.0,103.0]	<0.001	Kruskal-Wallis
Glucose, median [Q1,Q3]		108	107.0 [93.5,131.0]	98.5 [88.0,115.1]	0.007	Kruskal-Wallis
Free hemoglobin, median [Q1,Q3]		187	4.1 [2.5,13.5]	2.9 [2.2,4.4]	0.007	Kruskal-Wallis
Triglycerides, median [Q1,Q3]		61	113.0 [81.0,158.0]	93.0 [68.0,140.7]	0.011	Kruskal-Wallis
Mean cell hemoglobin (MCH), median [Q1,Q3]		19	29.7 [28.3,31.1]	30.2 [29.0,31.6]	0.011	Kruskal-Wallis
Potassium, median [Q1,Q3]		29	4.3 [4.0,4.6]	4.4 [4.2,4.7]	0.018	Kruskal-Wallis
Mean cell volume, median [Q1,Q3]		19	88.3 [84.9,91.8]	89.8 [86.1,93.9]	0.018	Kruskal-Wallis
Aspartate Aminotransferase , median [Q1,Q3]		18	25.0 [20.0,30.0]	28.0 [21.5,34.0]	0.018	Kruskal-Wallis
Serum urea , median [Q1,Q3]		15	18.4 [14.2,26.1]	21.1 [15.7,29.7]	0.019	Kruskal-Wallis
Calcium, median [Q1,Q3]		19	2.3 [2.3,2.4]	2.3 [2.2,2.4]	0.022	Kruskal-Wallis
Albumin, median [Q1,Q3]		90	41.7 [39.3,44.2]	40.5 [37.6,43.4]	0.022	Kruskal-Wallis
Lactate dehydrogenase , median [Q1,Q3]		30	205.0 [176.8,246.2]	217.0 [189.0,268.0]	0.024	Kruskal-Wallis
Billirubin, median [Q1,Q3]		21	0.5 [0.4,0.8]	0.7 [0.4,0.9]	0.024	Kruskal-Wallis
Alkaline phosphatase, median [Q1,Q3]		18	72.0 [56.0,92.0]	79.0 [59.5,106.8]	0.027	Kruskal-Wallis
Red blood cell count, median [Q1,Q3]		19	4.5 [4.0,4.8]	4.3 [3.9,4.7]	0.041	Kruskal-Wallis
Lymphocyte count (absolute), median [Q1,Q3]		54	1.6 [1.2,2.0]	1.3 [1.1,1.9]	0.052	Kruskal-Wallis
Thyroid-stimulating hormone (TSH), median [Q1,Q3]		126	1.7 [1.1,2.6]	2.1 [1.2,3.4]	0.062	Kruskal-Wallis
White blood cell count, median [Q1,Q3]		20	7.3 [5.9,8.9]	6.7 [5.6,8.5]	0.108	Kruskal-Wallis
Lipase, median [Q1,Q3]		135	32.0 [23.0,44.0]	35.0 [24.0,51.8]	0.176	Kruskal-Wallis
Monocyte count (relative), median [Q1,Q3]		54	7.9 [6.6,9.7]	8.6 [6.8,10.3]	0.176	Kruskal-Wallis
Neutrophil count (absolute), median [Q1,Q3]		54	4.7 [3.7,6.0]	4.4 [3.3,6.0]	0.286	Kruskal-Wallis
Magnesium, median [Q1,Q3]		48	0.8 [0.8,0.9]	0.8 [0.8,0.9]	0.286	Kruskal-Wallis
Uric acid, median [Q1,Q3]		45	6.4 [5.2,7.8]	6.5 [5.5,8.2]	0.288	Kruskal-Wallis
C-reactive protein , median [Q1,Q3]		35	0.3 [0.1,0.8]	0.3 [0.1,0.6]	0.288	Kruskal-Wallis
Platelet count, median [Q1,Q3]		19	225.0 [187.8,263.0]	217.0 [171.0,260.0]	0.468	Kruskal-Wallis
Eosinophil count (absolute), median [Q1,Q3]		54	0.1 [0.1,0.2]	0.1 [0.1,0.2]	0.469	Kruskal-Wallis
Mean platelet volume, median [Q1,Q3]		28	10.6 [10.0,11.3]	10.7 [10.2,11.4]	0.519	Kruskal-Wallis
Sodium, median [Q1,Q3]		15	140.0 [138.0,141.0]	139.2 [137.0,141.1]	0.519	Kruskal-Wallis
Hematocrit, median [Q1,Q3]		19	38.8 [35.6,42.5]	39.2 [34.2,42.1]	0.540	Kruskal-Wallis
Iron, median [Q1,Q3]		136	68.0 [46.0,99.5]	65.5 [45.0,92.5]	0.680	Kruskal-Wallis
Lymphocyte count (relative), median [Q1,Q3]		54	22.6 [16.1,28.2]	21.7 [16.8,27.8]	0.737	Kruskal-Wallis
Alanine transminase , median [Q1,Q3]		16	22.0 [17.0,29.5]	23.0 [17.0,31.6]	0.750	Kruskal-Wallis
LDL/HDL - cholestorol ratio, median [Q1,Q3]		175	3.5 [2.9,4.3]	3.3 [2.7,4.5]	0.897	Kruskal-Wallis
Basophile count (absolute), median [Q1,Q3]		54	0.0 [0.0,0.0]	0.0 [0.0,0.0]	0.897	Kruskal-Wallis
Alpha - amylase, median [Q1,Q3]		121	57.0 [42.0,76.0]	56.0 [40.0,75.4]	0.897	Kruskal-Wallis

train-cohort-lab-values

**Hemoglobin, median [Q1,Q3]**		21	13.1 [11.6,14.3]	13.2 [11.4,14.3]	0.897	Kruskal-Wallis
**Neutrophil count (relative), median [Q1,Q3]**		54	66.5 [59.4,72.7]	66.7 [60.3,73.6]	0.915	Kruskal-Wallis
**HDL – cholestorol, median [Q1,Q3]**		177	50.0 [39.8,60.0]	49.5 [38.2,60.8]	0.915	Kruskal-Wallis
**Eosinophil count (relative), median [Q1,Q3]**		54	1.8 [1.1,2.7]	1.8 [1.0,3.1]	0.915	Kruskal-Wallis
**Basophile count (relative), median [Q1,Q3]**		54	0.4 [0.3,0.6]	0.4 [0.3,0.6]	0.915	Kruskal-Wallis
**Creatine kinase , median [Q1,Q3]**		24	88.0 [63.0,141.0]	93.0 [62.0,133.0]	0.924	Kruskal-Wallis
**Cholesterol, median [Q1,Q3]**		62	176.0 [145.0,203.0]	168.0 [142.8,212.2]	0.924	Kruskal-Wallis
**Monocyte count, median [Q1,Q3]**		54	0.6 [0.5,0.7]	0.6 [0.5,0.7]	0.924	Kruskal-Wallis
