# Supplementary material for: Machine Learning Enables Prediction of Cardiac Amyloidosis by Routine Laboratory Parameters: A Proof-of-Concept Study
Source: J Clin Med. 2020 May 3;9(5):1334. doi: 10.3390/jcm9051334 (PMC7290438; doi:10.3390/jcm9051334)
Supplement: Supplementary file 1 [file jcm-09-01334-s001.zip › ttr-vs-al.pdf]

Supplementary Table 2. Baseline characteristics for all cardiac amyloidosis patients.

| | AL Amyloidosis (n=63) | Amyloidosis TTR wildtype (n=82) | Amyloidosis TTR variant (n=12) |
|---|---|---|---|
| **Variable, median [Q1-Q3]** | | | |
| Age, years | 66.0 [55.2,74.0] | 78.0 [73.2,82.8] | 65.0 [60.9,71.8] |
| NT-proBNP, pg/mL | 3904.0 [1648.8,9205.9] | 2815.0 [1409.3,5509.0] | 1488.0 [975.1,2499.5] |
| **Variable, mean (SD)** | | | |
| Body mass index, $kg/m^2$ | 26.1 (5.0) | 26.2 (3.6) | 24.0 (3.4) |
| **Variable, n (%)** | | | |
| Gender, males | 38 (60.3) | 69 (84.1) | 8 (66.7) |
| Coronary artery disease | 9 (14.3) | 27 (34.2) | 3 (25.0) |
| Atrial fibrillation | 15 (24.2) | 47 (58.0) | 3 (25.0) |
| Arterial hypertension | 35 (56.5) | 53 (66.2) | 4 (36.4) |
| Diabetes mellitus | 6 (9.7) | 13 (16.0) | 0 (0.0) |
| Hyperlipidemia | 15 (24.2) | 26 (32.9) | 1 (9.1) |
| MRA | 22 (37.3) | 39 (50.0) | 9 (75.0) |
| Calcium channel blocker | 4 (6.6) | 9 (11.2) | 0 (0.0) |
| Beta blocker | 29 (50.0) | 42 (55.3) | 5 (41.7) |
| Diuretics | 42 (68.9) | 57 (73.1) | 9 (81.8) |
| ACEI/ARB | 27 (44.3) | 39 (51.3) | 6 (54.5) |
| Oral anticoagulant | 22 (37.9) | 47 (58.0) | 4 (33.3) |
| Statin | 9 (14.8) | 35 (43.8) | 1 (9.1) |

NT-proBNP, N-terminal prohormone of brain natriuretic peptide; MRA, mineralocorticoid receptor antagonist; ACEI/ARB, angiotensin-converting enzyme inhibitors/angiotensin receptor blocker.
